# First insight into extracellular vesicle-miRNA characterization in a sheep *in vitro* model of inflammation

**DOI:** 10.3389/fvets.2023.1186989

**Published:** 2023-11-22

**Authors:** Maria Giovanna Ciliberti, Antonella Santillo, Agostino Sevi, Marzia Albenzio, Vincenzo De Leo, Chiara Ingrosso, Lucia Catucci, Mariangela Caroprese

**Affiliations:** ^1^Department of Agriculture, Food, Natural Resources, and Engineering (DAFNE), University of Foggia, Foggia, Italy; ^2^Department of Chemistry, University of Bari Aldo Moro, Bari, Italy; ^3^Department of Chemistry, Institute for Chemical and Physical Processes of National Research Council (CNR-IPCF), University of Bari, Bari, Italy

**Keywords:** biomarkers, LPS, cell model, livestock, immune system, small ruminant

## Abstract

Extracellular vesicles (EVs) and their microRNA (miRNA) cargoes have garnered attention in the veterinary field for their regulatory role in various biological processes. This study aimed to (i) evaluate two techniques of EV isolation from sheep peripheral blood mononuclear cell (PBMC) supernatants using the ultracentrifugation (UC) and reagent (REA) methods and (ii) characterize the EV-miRNA profiles after an *in vitro* inflammatory environment mediated by lipopolysaccharides (LPS). Sheep peripheral blood was collected, and PBMCs were separated using a density gradient reagent. Subsequently, PBMCs were cultured at 37°C for 24 h (5% CO_2_), and the supernatants were collected to perform the EV isolation. The presence of CD81^+^ extracellular vesicle marker was determined, and the purity of isolated EVs was calculated as a ratio between the number of isolated EVs and the protein concentration. Moreover, the morphological characterization revealed mainly round-shaped structures with average sizes of 211 nm for EVs isolated by the UC method and 99 nm for EVs isolated by the REA method. Illumina NextSeq sequencing in a single-end mode was used to characterize the miRNA profile, and the differentially expressed (DE) miRNAs were analyzed using a combination of bioinformatics tools. The results revealed that the REA method is reliable for EV isolation from sheep supernatants. It was considered an improvement of the recovery rate and purity of EVs with the enhancement of the number and the expression levels of characterized miRNAs. The EVs isolated by the UC method after an LPS challenge showed 11 DE miRNAs, among which eight miRNAs were upregulated and three were downregulated. On the other hand, the REA method revealed an EV cargo in which eight DE miRNAs were upregulated and 21 DE miRNAs were downregulated. The master miRNA regulators of the biological process were identified by performing the MIRNA-mRNA network analysis, showing that, among the higher representative miRNAs based on the centrality and betweenness, the miR-26a-5p could have a crucial role in the resolution of inflammation. Moreover, the identification of the let-7 miRNA family in all the EVs showed potential targeted genes that regulate the inflammation and immune responses.

## 1 Introduction

Recent studies in veterinary sciences have been showing a growing interest in extracellular vesicles (EVs) and their related molecular cargoes, particularly microRNAs (miRNA), which are considered potential disease biomarkers and therapeutic targets (1–4), given their regulatory role in various biological processes (5). EVs are defined as nanoparticles released by multiple cell types, and their biogenesis pathways include the endosome origin, with the release of exosome, and the plasma membrane-derived origin, with the release of microparticles/microvesicles (6). EVs are classified according to their size as small EVs (<200 nm) or medium-large EVs (>200 nm) (6). In a recent review by Moccia et al. (7), the potential use of EVs as markers to diagnose diseases or as possible natural transporters of therapies or vaccines in veterinary studies is discussed. Indeed, EVs are largely involved in immune and cell–cell communication and facilitate the transfer of DNAs, mRNAs, microRNAs, and lipids to both nearby and distant recipient cargo cells in relation to the onset and development of many diseases (8, 9).

MiRNAs are described as small non-coding RNA (ncRNA) molecules of approximately 22 nucleotides in length that have a principal role in the post-transcriptional process as a gene repressor or a destroyer of targeted mRNA by binding with the complementary base pair on 3′UTR, 5′UTR, or seeding and coding regions of targeted mRNA in plants, animals, and viruses (10, 11). A large number of protein-coding genes are under the control of miRNA, which is tightly regulated (10). Notably, miRNA species have been found to be significantly more abundant in EVs than in the cell of origin (12). During the pathogen invasion or under injuries, miRNAs can play a pivotal role in the activation of immune and physiological processes that are important for the removal of pathogens and the maintenance of homeostasis. Excitingly, miRNAs have the ability to regulate the secretion of immune mediators by the immune cells together with the maturation and differentiation of dendritic cells, macrophages, granulocytes, and other immune cells. Additionally, during inflammation in the bone marrow, miRNAs also play a role in developmental processes (13). Indeed, during inflammation and activation of the immune system, a class of miRNAs can work as important negative feedback loops in the immune system, whereas other miRNAs can be crucial for the amplification of the immune system’s response through repressing inhibitors of the response (13).

It is worth noting that the identification of the optimal technique to isolate EVs is essential in the purpose of searching for a novel biomarker. However, one of the main challenges in advancing our knowledge of EV functions is the lack of an efficient and standardized isolation strategy for isolating specific subpopulations and the absence of a gold standard method for isolating EVs (14). Therefore, the International Society for Extracellular Vesicles (ISEV) proposed the Minimal Information for Studies of EV (MISEV) guidelines in 2014 (14), upgraded in 2018, for improving EV research quality, recommending that there is no single optimal separation method; however, the choice ultimately depends on the specific downstream application and scientific question. Chen et al. (15) reviewed the common EV separation techniques, demonstrating that each separation strategy has both advantages and disadvantages. As the most common method, ultracentrifugation is a mature technology that can be used for separating most samples at low operating expenses. However, it is considered time-consuming, with poor/unstable repeatability, and may lead to a possible co-purification with protein aggregates, which may affect the results of subsequent mass spectrometry or protein quantitation (16–18). Furthermore, high-speed centrifugation may cause damage to EVs and reduce their biological activity (16). On the contrary, the isolation method based on kits is an “easy and quick” procedure that can suffer from co-isolation among EVs; however, it can be considered an ideal choice for the identification of exosome-related disease biomarkers (19).

In the present study, based on the assumption that isolation methods could affect EV cargo, two methods were selected for EV isolation from sheep peripheral blood mononuclear cell (PBMC) supernatants to compare the miRNomic profile after an *in vitro* inflammatory challenge mediated by LPS.

Hence, the present study aimed to (i) isolate EVs from sheep PBMC supernatants by using ultracentrifugation (UC) and total exosome isolation reagent (REA) methods and (ii) characterize the miRNomic profile after an inflammatory *in vitro* challenge, as a sheep model of inflammation. To the best of our knowledge, this is the first study that isolated EVs from sheep PBMC supernatant to analyze the miRNomic profile. Our hypothesis was focused on a potential connection between miRNAs, inflammation, and immune responses in sheep to define new predictive biomarkers associated with health or production phenotypes.

## 2 Materials and methods

### 2.1 Animals and experimental treatments

The Gentile di Puglia dairy sheep breed reared in the grazing system was used in this study (*n* = 3). All procedures were conducted according to the guidelines of the EU Directive 2010/63/EU (2010) on the protection of animals used for experimental and other scientific purposes. The animals were carefully examined by veterinarians throughout the trial to exclude the presence of any signs of disease (N. 12,917 del 20220303 2022-UNFGCLE-0012917).

### 2.2 Peripheral PBMC isolation

Blood samples (15 mL) were collected in Na-heparinized vacuum tubes from the jugular vein of sheep through a 21G needle, and the PBMCs were isolated by Histopaque®-1077 density gradient (Sigma Aldrich, Milan, Italy) according to Wattegedera et al. (20) and modified as previously reported by Ciliberti et al. (21). Briefly, whole blood diluted at a 1:1 ratio with cold PBS was centrifuged, and the white cell rings recovered after centrifugation were diluted in Hanks Balanced Salt Solution (HBSS, Thermo Fisher Scientific, Waltham, United States) and slowly layered on the Histopaque®-1077 solution (10 mL, Sigma Aldrich, Milan, Italy). The tubes were centrifuged at 400 × *g* for 30 min at 20°C, and the buffy coat containing the PBMCs layered on the upper layer of Ficoll-Paque was recovered. The PBMC suspension was washed three times with HBSS wash buffer containing 2% fetal bovine serum (FBS) exosome-depleted (Thermo Fisher, Waltham, United States) and penicillin/streptomycin mix antibiotics (Biowest, Riverside, United States). Finally, the PBMCs were resuspended in RPMI 1640 medium without calcium and magnesium (Sigma Aldrich, Milan, Italy), containing 10% FBS, penicillin/streptomycin antibiotics, and L-Glutamine solution. The PBMCs were counted using the trypan blue exclusion method on a Countess™ II Automated Cell Counter (Thermo Fisher Scientific, Waltham, United States). The viability of cells obtained after the PBMC isolation was higher than 98%. A final concentration of 1 × 10^6^ cells/mL was seeded into six well plates and cultured for 24 h at 37°C. The PBMCs were either stimulated with lipopolysaccharides (LPS) from *Escherichia coli* (1 μg/mL) or left unstimulated (CON).

### 2.3 Extracellular vesicle isolation from PBMC supernatants

**Figure 1:**
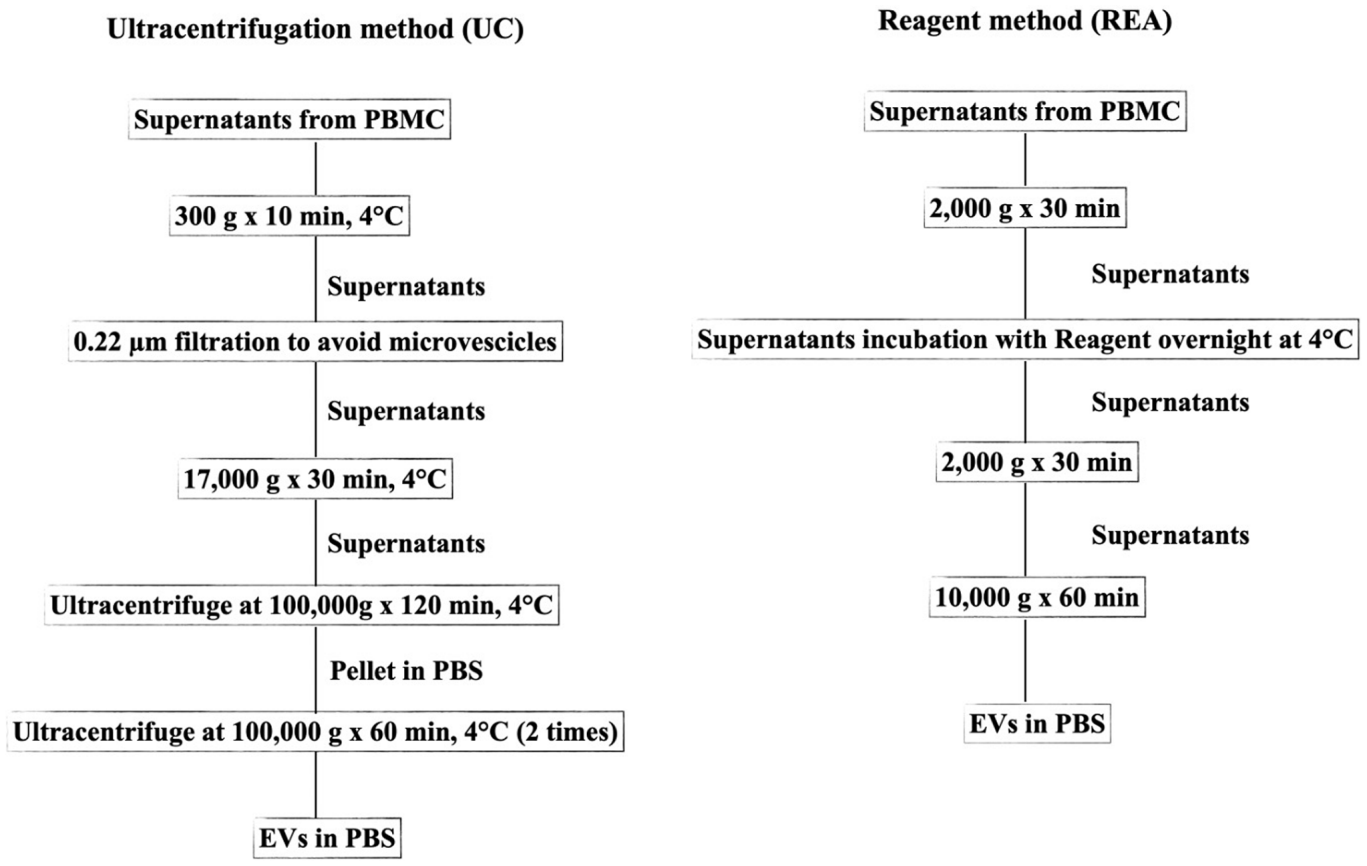
Flowchart representing the two methods (UC, Ultracentrifugation; REA, Reagent) used for EV isolation from the supernatant of sheep PBMCs.

After the supernatant free-cells collection from PBMCs, the EV isolation was performed using two different methods as depicted in the flowchart of the experiment (Figure 1).

The UC method was conducted according to Baharlooi et al. (22) with some modifications. Briefly, the collected supernatants were centrifuged at 300 *g* for 5 min to avoid cell or cellular contamination. Subsequently, the supernatant was filtered using a 0.22 μm filter and centrifuged at 17,000 *g* for 30 min to remove microvesicles. The collected supernatant was then transferred to a polypropylene ultracentrifuge tube and subjected to first ultracentrifugation at 100,000 × *g* for 120 min at 4°C with an Optima LE-80 K ultracentrifuge (Beckman Coulter, United States). Afterward, the supernatant was removed, and the EV pellet was suspended in PBS. Furthermore, the sample was ultracentrifuged at 100,000 × *g* for 60 min. All centrifugation steps were performed at 4°C. Finally, the supernatant was discarded and the pellet containing EVs was suspended in PBS and stored at −20°C for further analysis. The REA method was performed according to the manufacturer’s instruction of Total Exosome Isolation Reagent (from cell culture media, Thermo Fisher, Waltham, United States), and the isolation ratio was based on binding the water molecule and forcing the less-soluble molecules (EVs) out of the solution, resulting in a concentration of intact EVs and avoiding the time-consuming ultra-centrifugation procedure.

The number of EVs isolated was determined using ExoELISA-ULTRA Complete Kit CD81 detection (SBI System Bioscience, Embarcadero Way Palo Alto, CA, United States), and a standard curve was generated to determine the number of EVs positive to tetraspanin CD81 (CD81^+^) following the manufacturers’ recommendations.

Both the REA method and the Elisa kit used to count the final number of isolated EVs were calibrated on the NanoSight LM10 instrument as reported in the respective manufacturer’s instructions. The isolated EVs were analyzed for the protein content after lysis in RIPA buffer following the procedure described by Subedi et al. (23). The protein concentration was determined using the BCA Protein Assay kit (Thermo Scientific, Waltham, United States) according to the instruction manual, calibrated against the bovine serum albumin as a standard.

### 2.4 Zeta potential analysis and transmission electron microscopy

The zeta potential of EVs was measured by laser Doppler electrophoresis (LDE) with a Nanosizer ZS (Malvern Instruments, Malvern, United Kingdom), as previously reported by De Leo et al. (24).

Transmission electron microscopy analyses were performed by a Jeol Jem-1011 microscope (JEOL USA, Inc., Pleasanton, CA, United States) operating at 100 kV, equipped by a high-contrast objective lens, and a W filament as an electron source, with an ultimate point resolution of 0.34 nm. Images were acquired by a Quemesa Olympus CCD 11 MP camera. The samples were prepared by casting 3 μL of the aqueous dispersions of the isolated EVs onto 300 mesh amorphous carbon-coated Cu grids and then leaving the solvent to evaporate at room temperature. After deposition onto the grid, the samples were stained with a 2% aqueous solution of phosphotungstic acid hydrate. The size statistical analysis (EV average size and size distribution) of each sample (CON EVs isolated by UC and REA methods, and LPS EVs isolated using UC and REA methods) was performed on 100 nanostructures using a freeware Image J analysis program (National Institutes of Health, United States). The results are reported as mean ± standard deviation.

### 2.5 Library preparation and next-generation sequencing

The small RNA-Seq kit [Bioo Scientific NextflexTM (v2, v3)] was used for the library preparation following the manufacturer’s instructions. Then, the libraries were sequenced in single-end mode on the Illumina NextSeq. Before further analysis, a quality check was performed on the raw sequencing data using the FastQC tool available on http://www.bioinformatics.babraham.ac.uk/projects/fastqc for high throughput sequencing data. The quality trimming and adapter removal were done using the selected bioinformatics tool sRNAbench, following the procedure recommended by Aparicio-Puerta et al. (25).

### 2.6 miRNA identification and differential expression analysis

The sequences detected were analyzed using sRNAtoolbox (25), to identify miRNA expression profiles using an *ovis aries* species data set contained in miRBase (version 22) (26). The expression files generated with sRNAbench listed all copies of miRNAs. The NOISeq-Sim method was used to determine the differently expressed miRNAs of CON vs. LPS of the two EV isolating methods, and the probability of being differently expressed was calculated and set at >0.85.

### 2.7 Statistical analysis and bioinformatics

Data on EV CD81^+^ marker and protein quantification were checked by normality tests and analyzed with a one-way ANOVA of SAS (27). The significance of the differences was assessed using the Tukey *post hoc* test for multiple comparisons and a *p* value lower than 0.05 was considered statistically significant. The data were presented as mean ± SEM.

R was used to create a matrix of all transcripts expressed in all samples with the corresponding read counts, and the Bioconductor package NOISeq (28, 29) was used to normalize the data using the RSEM method and then to perform the differential expression analysis using NOISeq-Sim (28, 29).

The data on downregulated and upregulated differentially expressed miRNAs were analyzed using a functional enrichment analysis tool (FunRich, http://funrich.org/index.html) fed with human miRNA homologs retrieved through miRbase (version 22.2, https://mirbase.org). The FunRich tool performs statistical analysis for different lists of proteins, peptide analysis, clustering, or more complex proteomic analysis (30). The cutoff of the enrichment analyses was set to <0.05. The miRNAs-gene target interaction network analysis was performed and visualized using the miRNet web-based platform (version 2.0, https://www.mirnet.ca/miRNet/home.xhtml), and the KEGG functional enrichment analysis was performed based on miRNet network analysis of the downregulated and upregulated EVs-miRNAs.

## 3 Results

### 3.1 EV quantification and characterization

**Figure 2:**
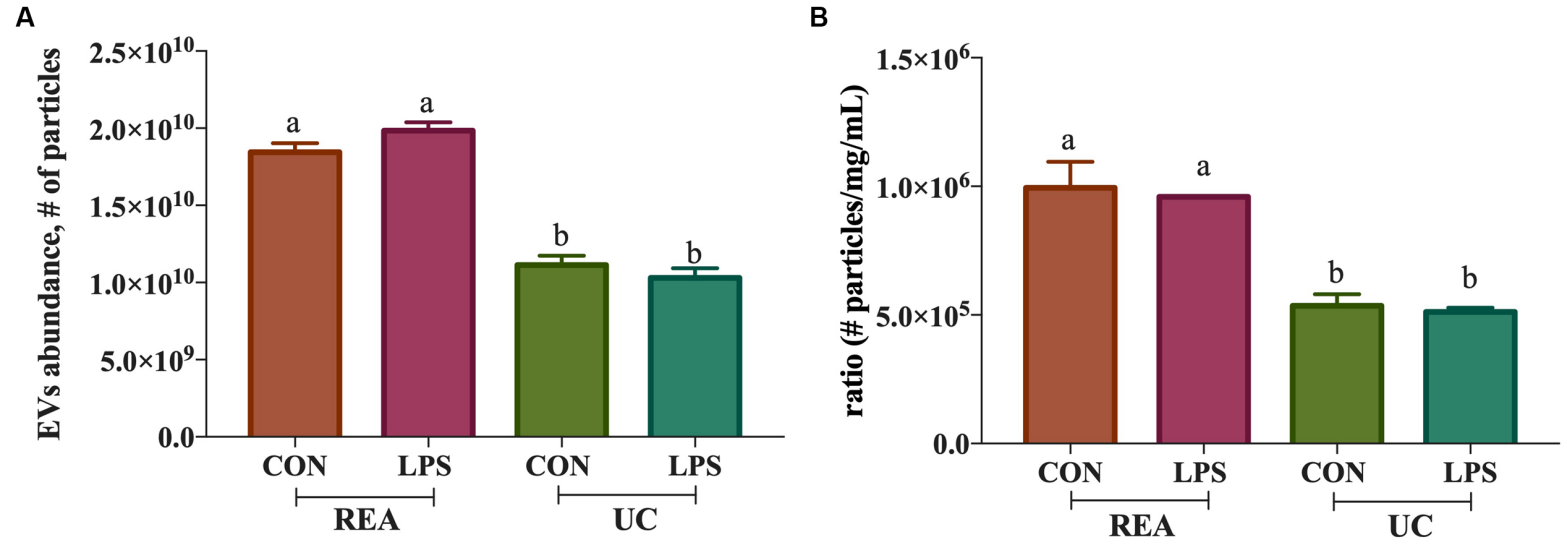
**(A)** Extracellular vesicle abundance (# of isolated extracellular vesicles ± SEM) using the two methods (UC, Ultracentrifugation; and REA, Reagent) in the presence and absence of LPS stimulus of inflammation; **(B)** Ratio of the number of extracellular vesicles isolated/protein concentration (mg/mL) of the ultracentrifugation (UC) and reagent (REA) methods in the presence and absence of LPS stimulus of inflammation. Data were presented as means ± SE.

**Figure 3:**
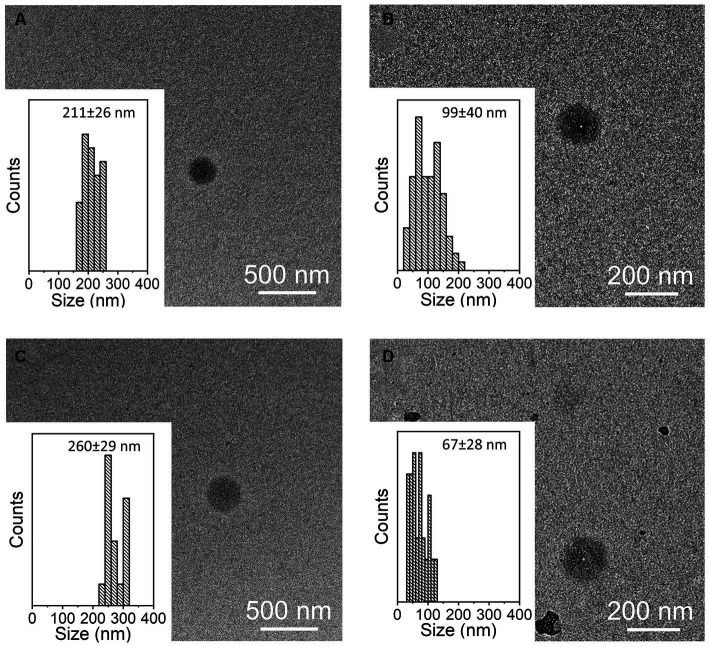
Representative TEM images of samples of extracellular vesicles (EVs) and size distribution measured on 100 EVs for each sample. **(A)** CON EVs isolated by the ultracentrifugation (UC) method; **(B)** CON EVs isolated by the reagent (REA) method; **(C)** LPS EVs isolated by the UC method; and **(D)** LPS EVs isolated by the REA method.

The presence of tetraspanin CD81^+^, located in the EV membrane, was used to calculate the number of isolated particles, showing a significant difference among treatments (*p* < 0.001, Figure 2A); indeed, the EV isolation performed using the REA method resulted in a higher quantity of EV isolation, apart from PBMC stimulation with LPS. Additionally, to calculate the purity of isolated EVs, the ratio of the number of EVs to protein concentration was calculated, as suggested by Tang et al. (31), and significant differences were found between the two isolation techniques, resulting in a higher ratio in the REA method than in the UC method (*p* = 0.006, Figure 2B). By means of TEM analysis, numerous round-shaped structures were clearly identified in the two types of samples, although slight polydispersion in particle size distribution emerged. In addition, a slight difference in the mean size of the EV samples was observed (Figure 3). The statistical analysis returned an average diameter value of 211 ± 26 nm for the EVs isolated with UC from the CON sample and 99 ± 40 nm for the EVs isolated with REA from the CON sample (Figures 3A,B). Stimulation with LPS did not result in significant changes in the observed dimensions, and the mean diameter was 260 ± 29 nm for EVs isolated by the UC method and 67 ± 28 nm for EVs isolated by the REA method (Figures 3C,D). Zeta potential values of all EV preparations were negative, as previously observed for EVs isolated from biological fluids (32). In particular, the measured values were − 12.3 ± 3.26 and − 27.2 ± 1.9 mV for the sample EVs from both UC and REA CON samples, respectively. Zeta potential values remained negative after stimulation with LPS, namely, −19.3 ± 6.8 mV for the EVs from the UC samples and 24.7 ± 0.78 mV for the EVs from the REA samples, suggesting good colloidal stability.

### 3.2 Differentially expressed miRNAs between isolation methods

**Figure 4:**
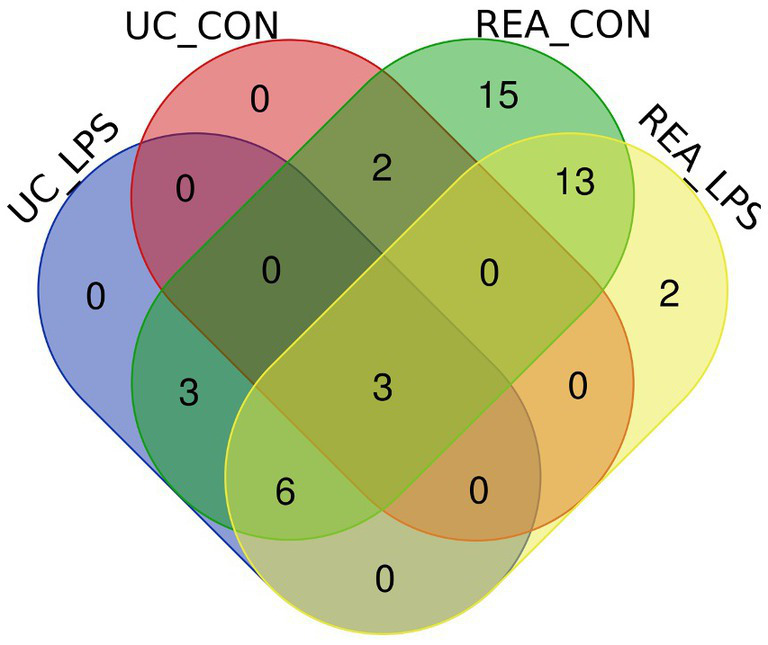
Venn diagram of extracellular vesicles-miRNAs characterized in PBMC supernatants by UC and REA methods and in the presence and absence of LPS stimulus of inflammation using the Funrich tool.

To compare the overlapping miRNAs between EV-miRNA cargoes from UC and REA isolation techniques, regardless of the presence of a stimulant, a Venn diagram was built (Figure 4). A total of five miRNAs for CON UC and 12 miRNAs for LPS UC were identified; the unique miRNAs found in REA CON and REA LPS were 42 and 24, respectively. The expression level for each mature miRNA characterized in all samples was reported in Supplementary Figure S1.

**Figure 5:**
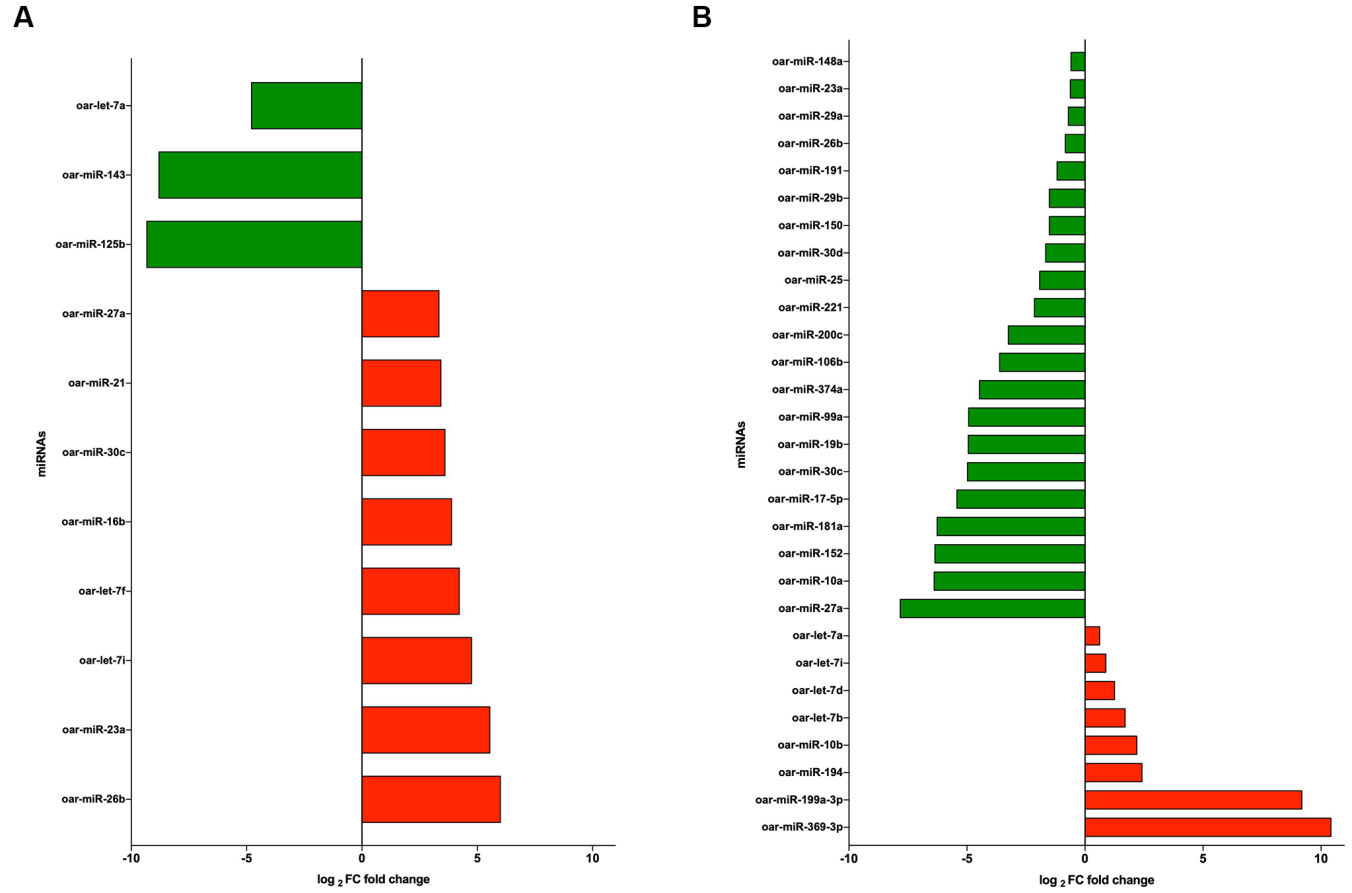
**(A)** log_2_ fold change of upregulated (green) and downregulated (red) miRNAs from extracellular vesicles (EVs) of CON vs. LPS isolated by the ultracentrifugation (UC) method. **(B)** log_2_ fold change of upregulated (green) and downregulated (red) miRNAs from EVs CON vs. LPS isolated by the Reagent (REA) method.

The significant differentially expressed (DE) miRNAs of EVs from PBMCs isolated by the UC method (CON vs. LPS) showed 11 DE miRNAs in total, among which eight miRNAs were upregulated (oar-miR-26b, oar-miR-23a, oar-let-7i, oar-let-7f, oar-miR-16b, oar-miR-30c, oar-miR-21, and oar-miR-27a), with fold change (FC) > 1.5 and probability > 0.85, and three were downregulated (oar-miR-125b, oar-miR-143, and oar-let-7a), with FC < 1.5 and probability > 0.85 (Figure 5A). On the contrary, using the REA method (CON vs. LPS) showed that among the DE miRNAs, eight were upregulated (oar-miR-369-3p, oar-miR-199a-3p, oar-miR-194, oar-miR-10b, oar-let-7b, oar-let-7d, oar-let-7i, and oar-let-7a), with FC > 1.5 and probability > 0.85 (Figure 5B), and 21 miRNAs were downregulated (oar-miR-27a, oar-miR-10a, oar-miR-152, oar-miR-181a, oar-miR-17-5p, oar-miR-30c, oar-miR-19b, oar-miR-99a, oar-miR-374a, oar-miR-106b, oar-miR-200c, oar-miR-221, oar-miR-25, oar-miR-30d, oar-miR-150, oar-miR-29b, oar-miR-191, oar-miR-26b, oar-miR-29a, oar-miR-23a, and oar-miR-148a), with FC < 1.5 and probability > 0.85 (Figure 5B). Among the DE miRNAs, the REA and UC methods had in common six miRNAs among which oar-miR-26b, oar-miR-30c, oar-miR-27a, and oar-miR-23a were upregulated in UC and downregulated in REA, the miRNA oar-let-7i was upregulated in both the UC and REA methods, and oar-let-7a was upregulated in the REA method and downregulated in the UC method.

### 3.3 Functional and pathway enrichment analysis for identified miRNA target genes

#### 3.3.1 Ultracentrifugation method

**Figure 6:**
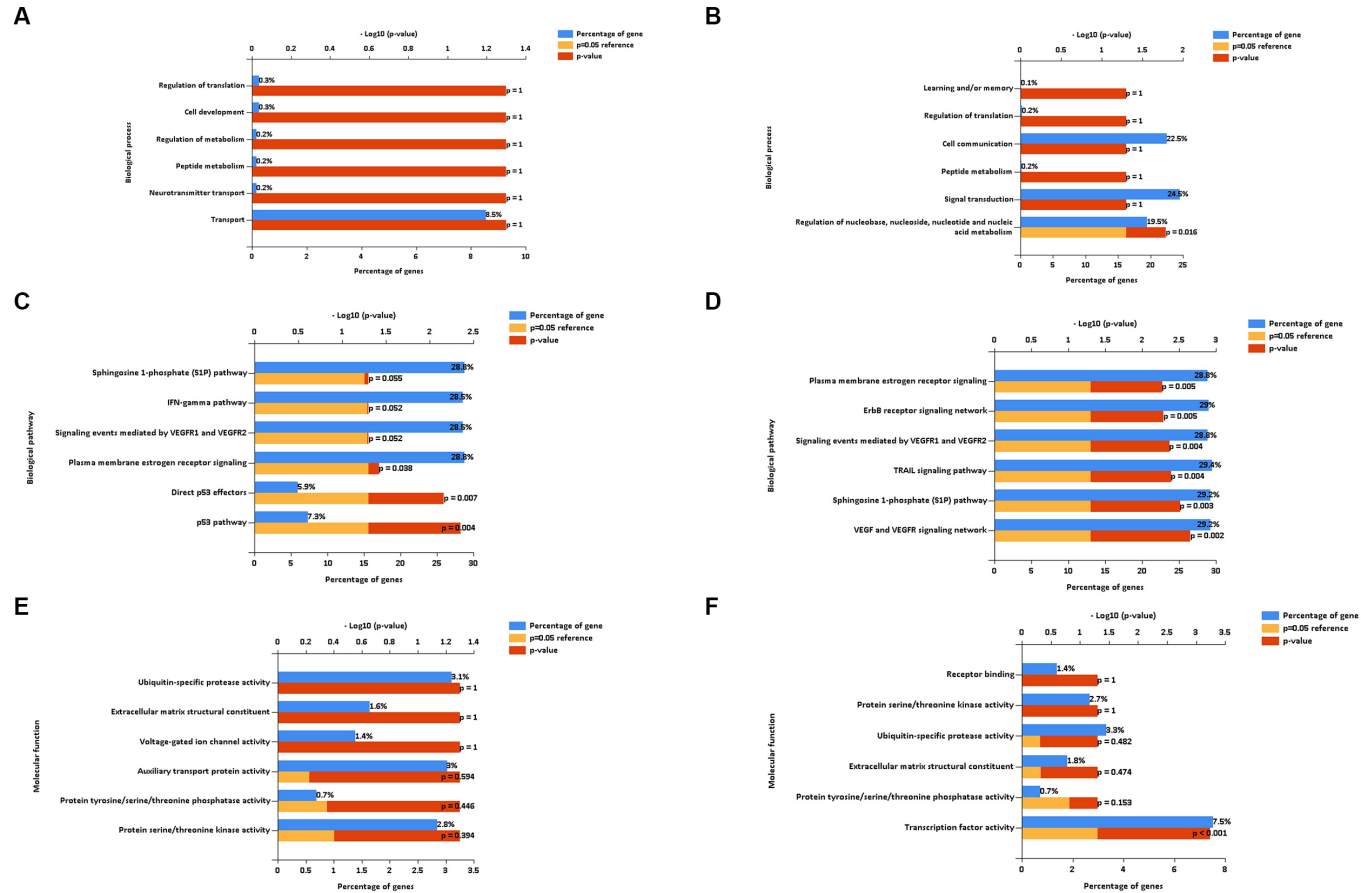
Biological process **(A,B)**, biological pathways **(C,D)**, and molecular function **(E,F)** of down and up regulated extracellular vesicles miRNAs isolated from PBMC supernatants by using ultracentrifugation (UC) method.

To gain a better understanding of the function and mechanism of miRNAs, we analyzed the data on the downregulated and upregulated DE miRNAs. We used the online enrichment tool Funrich to carry out this analysis, and the biological processes (BPs), biological pathways (BPath), and molecular functions (MFs) were analyzed using this tool. After the LPS challenge, the downregulated miRNAs did not result in any significantly enriched genes in the BP term (Figure 6A). On the contrary, the upregulated miRNAs resulted in enriched genes in the BP term related to the regulation of nucleobase, nucleoside, nucleotide, and nucleic acid metabolism (Figure 6B). Regarding the BPath term, the significant genes enriched for the downregulated miRNAs (Figure 6C) were implicated in the p53 pathway (7.3%, *p* = 0.004), direct p53 effectors (5.9%, *p* = 0.007), and plasma membrane estrogen receptor signaling (28.8%, *p* = 0.038). The upregulated miRNAs (Figure 6D) resulted in the six main representative significantly enriched genes related to plasma membrane estrogen receptor signaling (28.8%, *p* = 0.005), ErbB receptor signaling network (29%, *p* = 0.005), signaling events mediated by VEGFR1 and VEGFR2 (28.8%, *p* = 0.004), TRAIL signaling pathway (29.4%, *p* = 0.004), sphingosine 1-phosphate (S1P) pathway (29.2%, *p* = 0.003), and the VEGF and VEGFR signaling network (29.2%, *p* = 0.002).

Regarding the MF term, the downregulated miRNAs did not result in any significantly enriched genes (Figure 6E), whereas the upregulated miRNAs resulted in the enriched genes involved in the significant transcription factor activity (7.5%, *p* < 0.001, Figure 6F).

#### 3.3.2 Reagent method

**Figure 7:**
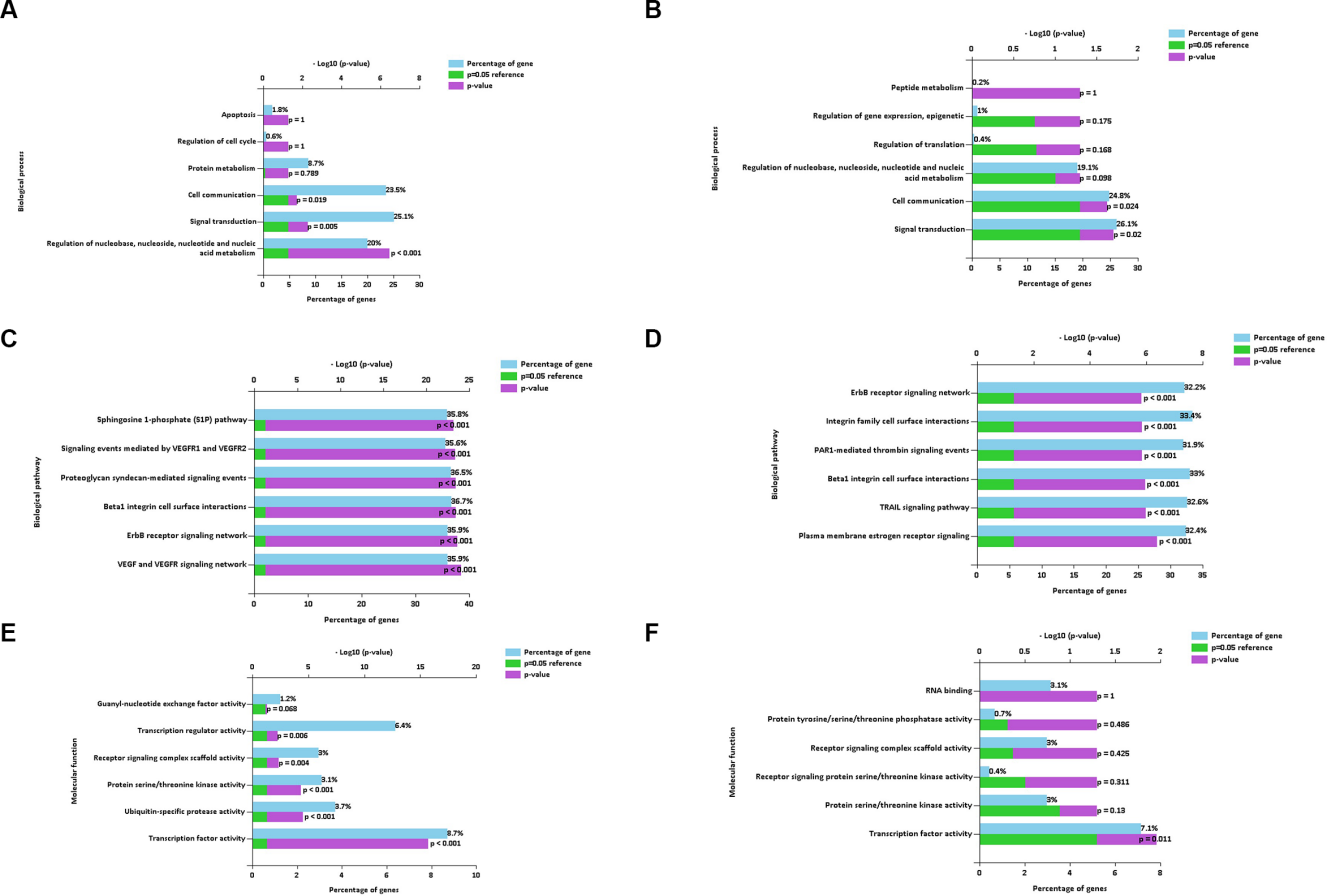
Biological process **(A,B)**, biological pathways **(C,D)**, and molecular function **(E,F)** of down and up regulated extracellular vesicles miRNAs isolated from PBMC supernatants by using Reagent (REA) method.

The main enriched genes involved in the downregulated (Figure 7A) and upregulated (Figure 7B) miRNAs of the BP terms characterized in EVs isolated using the REA method (CON vs. LPS) were implicated in cell communication and signal transduction. In addition, the enriched genes of the downregulated miRNAs were also implicated in the regulation of nucleobase, nucleoside, nucleotide, and nucleic acid metabolism of the BP term (20%, *p* < 0.001).

The enriched genes of the BPath terms involved in both downregulated (Figure 7C) and upregulated (Figure 7D), miRNAs were all significant (*p* < 0.001). In particular, the enriched genes with the highest percentages in both downregulated and upregulated miRNAs were implicated in the beta1 integrin cell surface interactions of the BPath term (33.7 and 33%, respectively).

The significantly enriched genes of the main MF term were related to the transcription factor activity for both downregulated (8.7%, *p* < 0.001, Figure 7F) and upregulated miRNAs (7.1%, *p* = 0.011, Figure 7E). Moreover, the enriched genes of the downregulated miRNAs were also implicated in the significant transcription regulator activity (6.4%, *p* = 0.006), followed by ubiquitin-specific protease activity (3.7%, *p* < 0.001).

### 3.4 Identification of extracellular vesicle miRNAs from PBMC

#### 3.4.1 miRNA-gene target interaction networks and KEGG pathways

**Figure 8:**
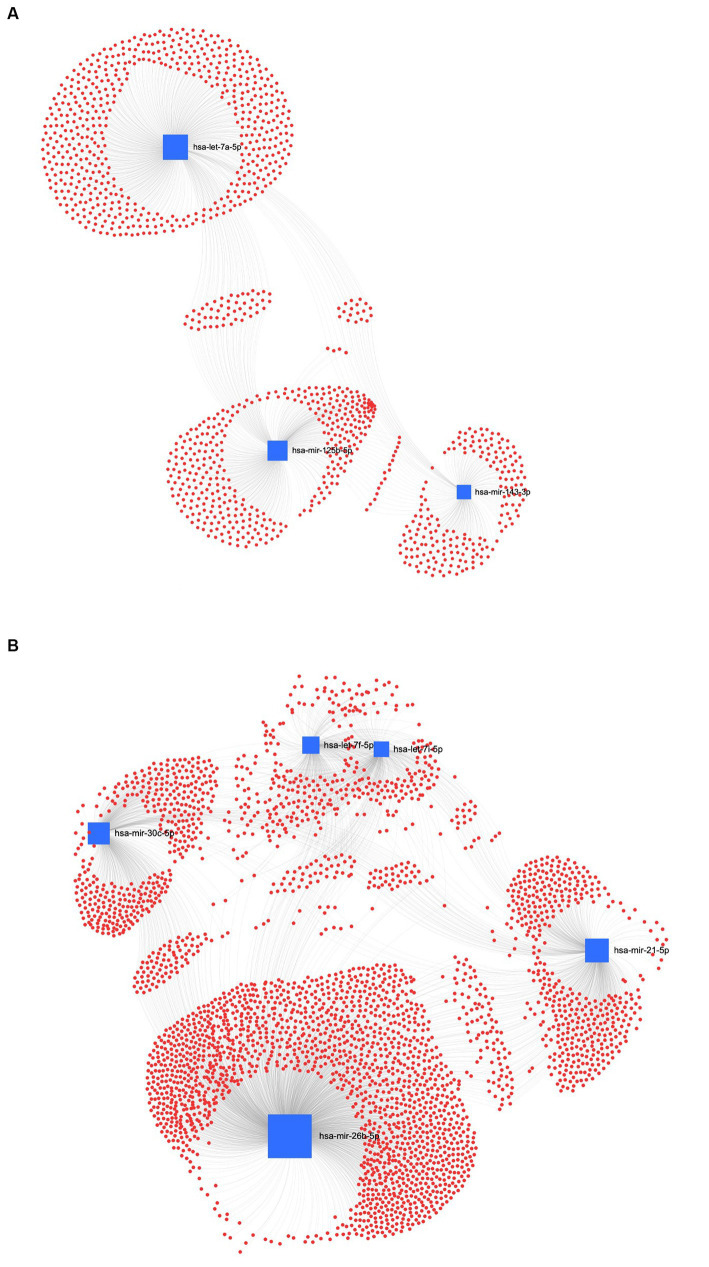
MiRNA-gene target network analysis. The centric target network of upregulated **(A)** and downregulated **(B)** target genes in ultracentrifugation (UC) samples of EVs by miRNet software. The boxes represent miRNAs (blue) with the size resulting from the betweenness of the miRNAs in constructing the network. The nodes represent the genes (red).

The interaction network between miRNAs and their potential target genes is crucial for identifying the biological relevance of miRNAs. Accordingly, in the UC method, Figure 8A shows the ForceAtlas of the downregulated EV human homolog miRNAs in which hsa-let-7a-5p had a higher centrality and betweenness than hsa-miR-125b-5p and hsa-miR-143-3p. Among the upregulated EVs-miRNAs from UC, the human homolog hsa-miR-26a-5p showed higher centrality and betweenness than hsa-let-7i-5p (Figure 8B).

**Figure 9:**
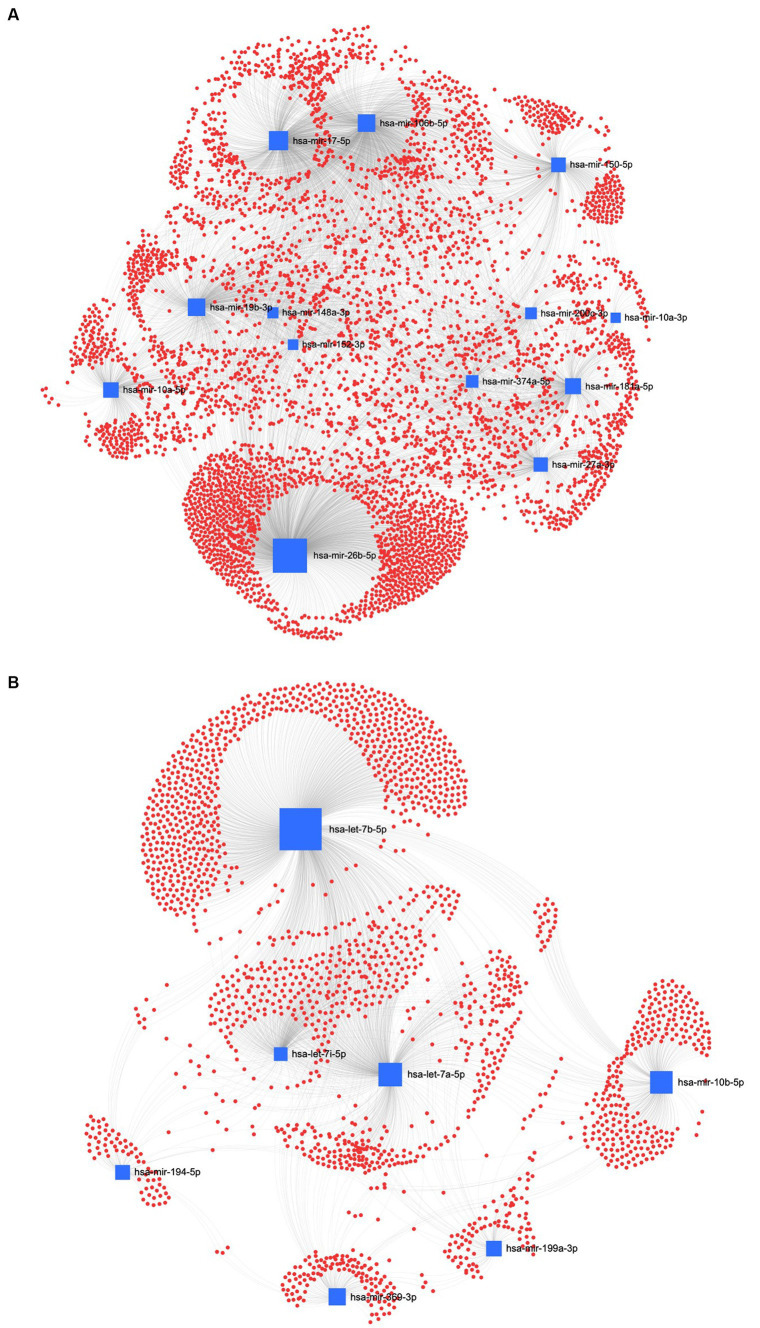
MiRNA-gene target network analysis. The centric target network of upregulated **(A)** and downregulated **(B)** target genes in reagent (REA) samples of EVs by miRNet software. The boxes represent the miRNAs (blue) with size resulting from the betweenness of the miRNAs in constructing the network. The nodes represent the genes (red).

In the REA method, the downregulated EVs-miRNAs of the human homologs, hsa-miR-26a-5p and hsa-miR-17-5p, had the highest centrality and betweenness (Figure 9A). Furthermore, among the upregulated miRNAs, hsa-let-7b-5p, hsa-let-7a-5p, and hsa-let-7i-5p resulted in higher centrality and betweenness than the other upregulated miRNAs (Figure 9B). To further explore the biological function of the predicted target genes, the KEGG pathway analysis of the significantly downregulated and upregulated miRNAs in both UC and REA samples was reported (Supplementary Figures S2, S3).

## 4 Discussion

In recent years, the putative role of miRNAs as biomarkers in farm animal diseases and as a therapeutic approach based on their regulatory key role in disease has been explored (33). To the best of our knowledge, this is the first study in which the characterization of the miRNA profile of EVs isolated from supernatants of sheep PBMCs, challenged *in vitro* with LPS, has been investigated. One of the most significant scientific challenges to date is the isolation and characterization of EVs. Understanding the importance of EVs-miRNAs is a crucial aspect of this research (14, 34). Therefore, the identification of the optimal technique to isolate EVs is crucial when the main purpose is the characterization of miRNAs for further biomarker discoveries. Particularly, in the present study, we focused on two methods of EV isolation starting from cell culture supernatants. EV isolation using the UC method is a traditional technique, but it requires prior training and can be quite tedious and time-consuming. Additionally, the results may be sensitive to the technique used (35). Our results demonstrated that the UC method decreases the recovery rate of CD81^+^ EVs, as also confirmed by the low purity calculated as the ratio of particle number to protein concentration. According to the studies conducted by Helwa et al. (35) and Lane et al. (36), the UC isolation method resulted in a lower recovery of EVs compared to commercially available kits. This result can be due to the different centrifugation forces over multiple cycles, useful for removing cell debris and other contaminants, which can also cause a loss of EVs from the sample, leading to a lower and more variable EV yield (37). According to the study conducted by Tang et al. (31), the number of EVs isolated using the UC method was lower compared to that obtained using two commercial kits specifically designed for EV isolation (ExoQuick and Total Exosomes Isolation Reagent_TEI). However, even if the UC method resulted in a lower number of EVs, more enriched EV markers by using Western blotting were obtained (31). In contrast with these findings, the use of a centrifuge at high speeds can affect the EVs’ physical properties and the sensitivity of the proteomic analysis (38, 39). Accordingly, in the present study, the EV-miRNA characterization revealed that the UC method reduces the EV-miRNA cargoes, in terms of the number of miRNAs identified, as depicted in the Venn diagram. This result is consistent with the lower EV recovery rate and purity due to the UC isolation method. To date, this study is the first on EV characterization from sheep PBMC supernatants; therefore, it will be a crucial step to confirm our data with additional EV protein characterization based on the methods reported in the MISEV2018 guidelines.

MiRNAs are recognized as playing a key role in regulating gene and biological processes such as cell proliferation, apoptosis, differentiation, and tumorigenesis (40, 41). Mature miRNAs regulate the expression of the target gene by recognizing and complementing completely or incompletely the target mRNA sequences through 5–8 nucleotides at its 5′ end and then inducing the degradation or translational inhibition of target mRNA; thus, mature miRNAs regulate the expression of the target gene (42).

In veterinary studies, miRNAs have been proven to mediate cellular immunity, apoptosis, signal transduction, and cell differentiation (43, 44). In particular, the initiation of inflammatory stimuli triggers both miRNAs and protein-coding genes. On the one hand, the expression of many miRNAs is regulated by silencers, enhancers, and epigenetic modification such as hypomethylation and hypermethylation in miRNA promoter and transcription factors (45, 46). Of these transcription factors, the nuclear factor-κB (NF-κB) is the one that regulates the expression of various inflammation-associated miRNAs (miR-146a and miR-155) (47–49) and functions as a strong pro-inflammatory signal (47), inducing the expression of proinflammatory genes, including cytokines and chemokines (50). In dairy cows, miRNAs can exert a post-transcriptional regulation of endocrine, metabolic, and immune reactions activated by the energy imbalance occurring during the transition to lactation (51, 52). In particular, in circulating miRNA profiles of transition dairy cows, a selection of miRNA subsets have been identified as putative biomarkers for metabolic disorders, with these biomarkers being the primary affected pathways linked to the metabolic and immune adaptation (52). In the present study, the EVs-miRNAs, characterized using both methods and analyzed considering the *in vitro* LPS challenge, showed a different subset to upregulated and downregulated miRNAs. Notably, the REA and UC methods had in common, among the DEM, the miR-26b, which was upregulated in the UC method and downregulated in the REA method. The miR-26 family has been recognized to target regulators that control the development (53), cell type, and differentiation of tumors (54). Zhu et al. (55) reported that, in both physiological and pathological conditions, miR-26b is expressed concomitantly with its host genes, cooperating to block the G1/S-phase transition of proliferation through the activation of the pRb protein (55). The overexpression of miR-26b was directly related to the composition of milk fatty acids in an *in vitro* study on goat mammary epithelial cells (56). In addition, the role of miR-26b was associated with numerous metabolic diseases, playing a crucial role in adipocyte development (56) and lipogenesis (57). The synthetic bta-miR-26b was found to downregulate three immune system-related genes in primary bovine endometrial epithelial cells (EECs); thus, bta-miR-26b has been a candidate miRNA among the potential miRNAs that regulate the immune system during peri-implantation periods (58).

The effect of miR-17-5p on inflammation mediated by LPS was tested in an *in vitro* study on RPMI2650 cells conducted by Huang et al. (59), who found that the overexpression of miR-17-5p intensifies the inflammation through the negative regulation of Smad7 protein expression, which exerts a protective role by the inactivation of NF-κB and Wnt/β-catenin pathways. Moreover, miR-17-5p acts as a key regulator of the G1/S cell cycle de-coupling negative regulators of the MAPK signaling cascade (60). In the present study, KEGG analysis revealed the most enriched pathways of the target genes of downregulated and upregulated miRNAs, the implication of the MAPK signaling pathway. The MAPK family has a central role in many physiological processes such as apoptosis and inflammation, with direct or indirect regulation of the NF-κB activity, which is the key transcription factor to promote the expression of inflammatory cytokines (61). The recruitment of the MAPK pathways is activated by pathogen-associated molecular patterns (PAMPs), molecular portions present in invading pathogens, and by damage-associated molecular patterns (DAMPs), molecules produced endogenously in times of physiological stress, which support a robust inflammatory response including cytokine production and leukocyte activation (62). In the present study, the implication of the MAPK pathway of the target genes of upregulated miRNAs could establish the relationship between the post-transcriptional information driven by EV-miRNAs and the response to the inflammation mediated by LPS. Previous studies stated that the cell injury induced by LPS can be mediated by the modulatory action of miRNAs (63). Moreover, results from the present study, from the interaction network between miRNAs and their potential target genes, suggest that both miR-26b and miR-17-5p could be considered among the master regulators of the biological processes activated by LPS. No previous studies have been conducted on sheep PBMC supernatant miRNA characterization; however, it could be hypothesized that miR-26b and miR-17-5p can have a crucial role in the inflammation process mediated by LPS in blood cells and in its resolution, even if further studies to better elucidate this hypothesis are needed.

From the interaction network between miRNAs and their potential target genes data analysis, it was found that there is a high number of let-7 family miRNAs present, among which oar-let-7a, oar-let-7b, oar-let-7d, and oar-let-7i emerged as the most prominent. In the study of Izumi et al. (64), the let-7a miRNA was found to be highly expressed in bovine raw milk exosomes (MEVs) and was also present in human and pig milk exosomes (65, 66). The highly expressed miRNAs of milk exosomes from several species exhibited common functions, such as regulating immune function and intestinal maturation (64). Indeed, the role of microRNAs in mammary gland development, health, and function of cattle, goats, and sheep was demonstrated (67). Moreover, metabolomic and transcriptomic approaches applied to MEVs from cows, donkeys, and goats revealed that numerous metabolic pathways implicated in immunomodulation (68) and miRNA targets with enriched terms related to the immunity modulation, protein synthesis, and cellular cycle regulation were common in the cow, donkey, and goat species (69).

The let-7f and let-7b miRNAs are highly expressed in both cow and sheep milk with established immune-related functions (70). The let-7b targets toll-like receptors (TLR), regulating the activation of NF-κB, and its downstream genes were related to the inflammation and immune responses during an *in vitro Helicobacter pylori* infection of human gastric epithelial cells (71). The miRNA profiling of sheep responding to the infection of small ruminant lentiviruses showed that DE miR-21, miR-148a, and let-7f regulated the seronegative and infected sheep, therefore demonstrating their potential implications for the host-virus interaction (72). Moreover, the let-7a miRNA targets the IL-6 gene, resulting in a downregulation of tumors related to an *in vitro* cell model of inflammation (73, 74). In the present study, the let-7 miRNA family was implicated under the LPS inflammatory challenge, showing both upregulated and downregulated miRNAs, thus demonstrating its regulatory biological function during inflammation in sheep PBMC. Moreover, a promising implication of the IL-6 gene may reveal a possible cross-link between inflammatory pathways and miRNAs, as recently reviewed by Chatterjee et al. (75), which needs to be further explored. Indeed, IL-6 is well known as a potential biomarker of inflammation both in human and ruminant studies (21, 76, 77).

From the KEGG analysis emerged the involvement of the p53 signaling pathway from the gene target of all characterized DE miRNAs. It has been suggested that the proper balance between the p53 and MAPK signaling pathways is necessary after a stressful stimulus is applied to the cells as this will determine whether the cells survive or die. The involvement of the p53 signaling pathway in this balance has been proposed as a potential link between the two pathways (78). Indeed, the activation of p53 causes growth arrest and apoptosis through the suppression of a number of genes that may favor cell survival (78). MiR-143 is classified as a tumor suppressor by arresting the G0/G1 phase cells and promoting caspase-3-apoptosis (79). Moreover, a specific apoptosis mechanism activated by miR-143 is involved in the targeting of MDM2 and, indirectly, in the activation of the p53 pathway, which in turn activates the transcription of miR-143, generating a short miR-143-MDM2-p53 feedback loop (18, 80, 81). Previous evidence could explain the characterized downregulation of oar-miR-143 in the UC sample and the concomitant augmentation of the biological p-53 pathway.

Additionally, the KEGG analysis showed that the most enriched pathways of the target genes of both upregulated and downregulated DE miRNAs from the UC and REA methods after LPS stimulation were associated with the JAK/STAT signaling pathway. In the study of Yang et al. (82), it was found that miR-203a-3p induces the polarization of macrophage-M2 by downregulating the suppressor of cytokine signaling 3 (SOCS3) expression and activating JAK/STAT3 signaling (82). It is well known that SOCS is a protein family of eight members (SOCS1–7 and CIS) that inhibit STAT activation by acting on JAK/STAT activating receptors. A previous study highlighted that miRNAs are involved in the regulation of innate immune and inflammatory responses (83). In ruminant studies, the nexus between circulating miRNAs and the cytokine profiles could be considered a key factor in studying their disease susceptibility. In a study conducted by Naylor et al. (84) on lambs challenged with LPS at a systematic level, it was observed that there was a simultaneous increase in both immune and stress biomarkers. Some of the biomarkers that showed an increase were IL-6, cortisol, and certain miRNAs, including miR-145, miR-1246, and miR-223. Moreover, it was demonstrated that, following LPS-mediated inflammation in a murine dendritic cell line derived from bone marrow, miR-369-3p can suppress the inflammation by reducing the secretion of proinflammatory molecules (TNFα, IL-6, IL-12, IL-1α, and IL-1β) while increasing the levels of IL-10 and IL-1RA, with anti-inflammatory roles (85). In the present study, miR-369-3p was upregulated in REA probably as a result of its suppressive action in inflammation mediated by LPS. In a study conducted by Gholami et al. (86), some miRNAs were involved in the initiation of inflammatory response by modulating many immune responses including the secretion of cytokines and chemokines such as miR-26a. Accordingly, to select potential miRNAs as novel biomarkers in sheep, it will be fundamental to understand the specific functions and the mechanisms of interaction between miRNAs and other biomolecules involved in disease pathogenesis, which include proinflammatory cytokines and plasma proteins (87).

## 5 Conclusion

In the present study, two different isolation methods were used to compare the EV-miRNome from sheep supernatant PBMCs. The data demonstrated that the REA method is reliable for EV isolation, enhancing the recovery rate and the purity of CD81^+^ EVs, and improving the number of miRNAs characterized. However, this study being the first on sheep supernatant EV characterization, additional methods for more in-depth protein characterization as reported in the MISEV2018 guidelines are going to be performed in the future.

Among the DE miRNAs, the EVs from PBMC isolated using the UC method displayed eight upregulated miRNAs and three downregulated miRNAs, while the REA EV isolation method was characterized by eight upregulated miRNAs and 21 downregulated miRNAs. Moreover, the tested isolation methods of the DE miRNAs had in common the presence of miR-26b and miR-17-5p, which could play a crucial role in the inflammation resolution mediated by LPS, as well as the let-7 miRNA family characterized in all the EV targeted genes that regulate the inflammation and immune responses.

The present study’s data can provide insights into the putative role of EVs-miRNAs in a sheep model of inflammation mediated by an LPS challenge.

## Data availability statement

The datasets generated for this study are available on request to the corresponding author.

## Ethics statement

The animal study was approved by Scientific Ethics Commitee of the University of Foggia. The study was conducted in accordance with the local legislation and institutional requirements.

## Author contributions

MCi, MCa, and AgS contributed to the conception and design of the study. MCi supervised the overall project and performed the statistical analysis. MCi, VL, CI, and LC performed the analysis. MCi and AnS organized the database. MCi, MA, AnS, and MCa wrote the first draft of the manuscript. VL, CI, and LC contributed to the revision of the manuscript. All authors contributed to the article and approved the submitted version.
