# Supplementary figures and images for: First insight into extracellular vesicle-miRNA characterization in a sheep in vitro model of inflammation
Source: Front Vet Sci. 2023 Nov 22;10:1186989. doi: 10.3389/fvets.2023.1186989 (PMC10703394; doi:10.3389/fvets.2023.1186989)

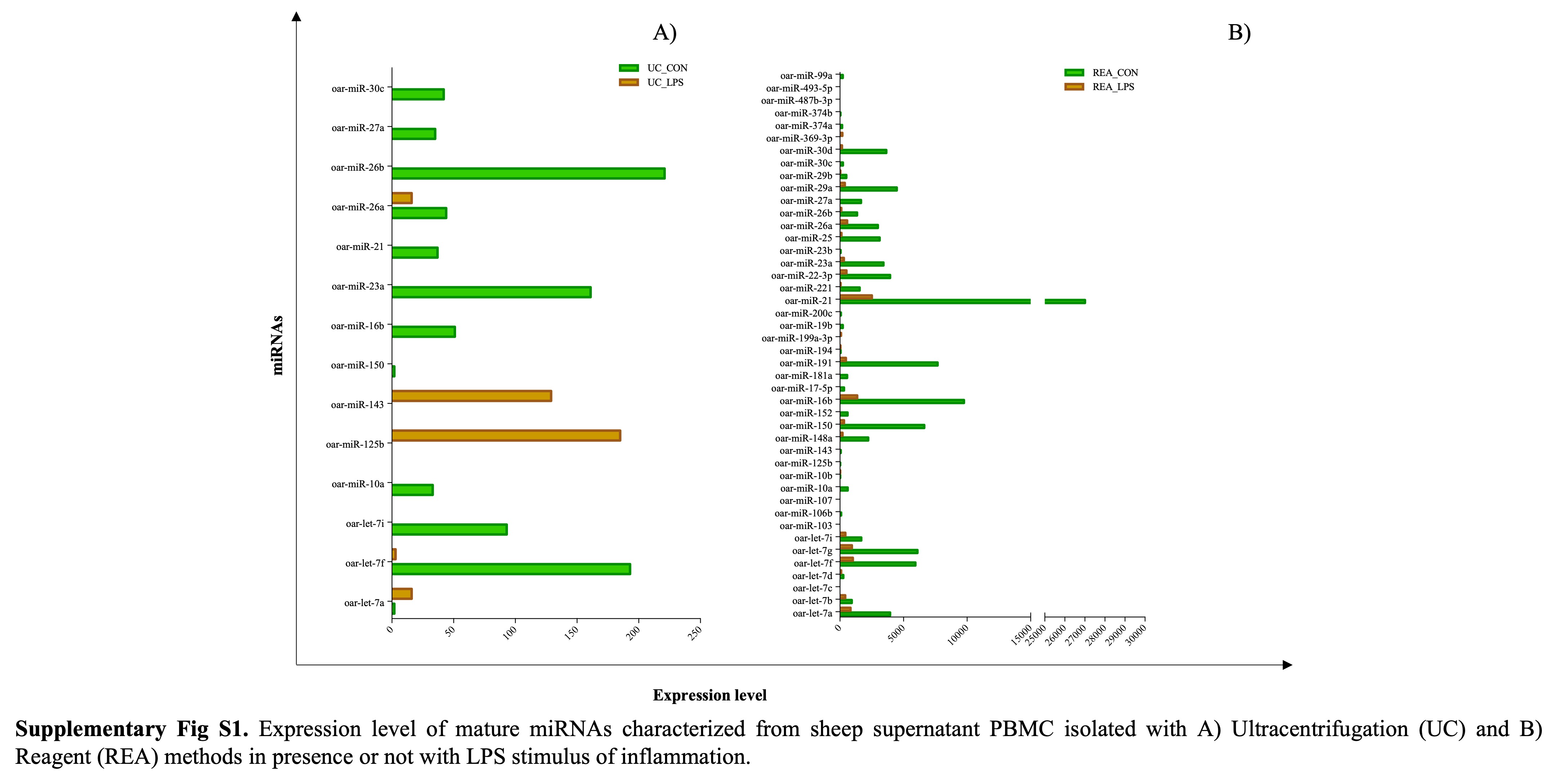

Supplement: Supplementary file 1 [file Image_1.jpg]

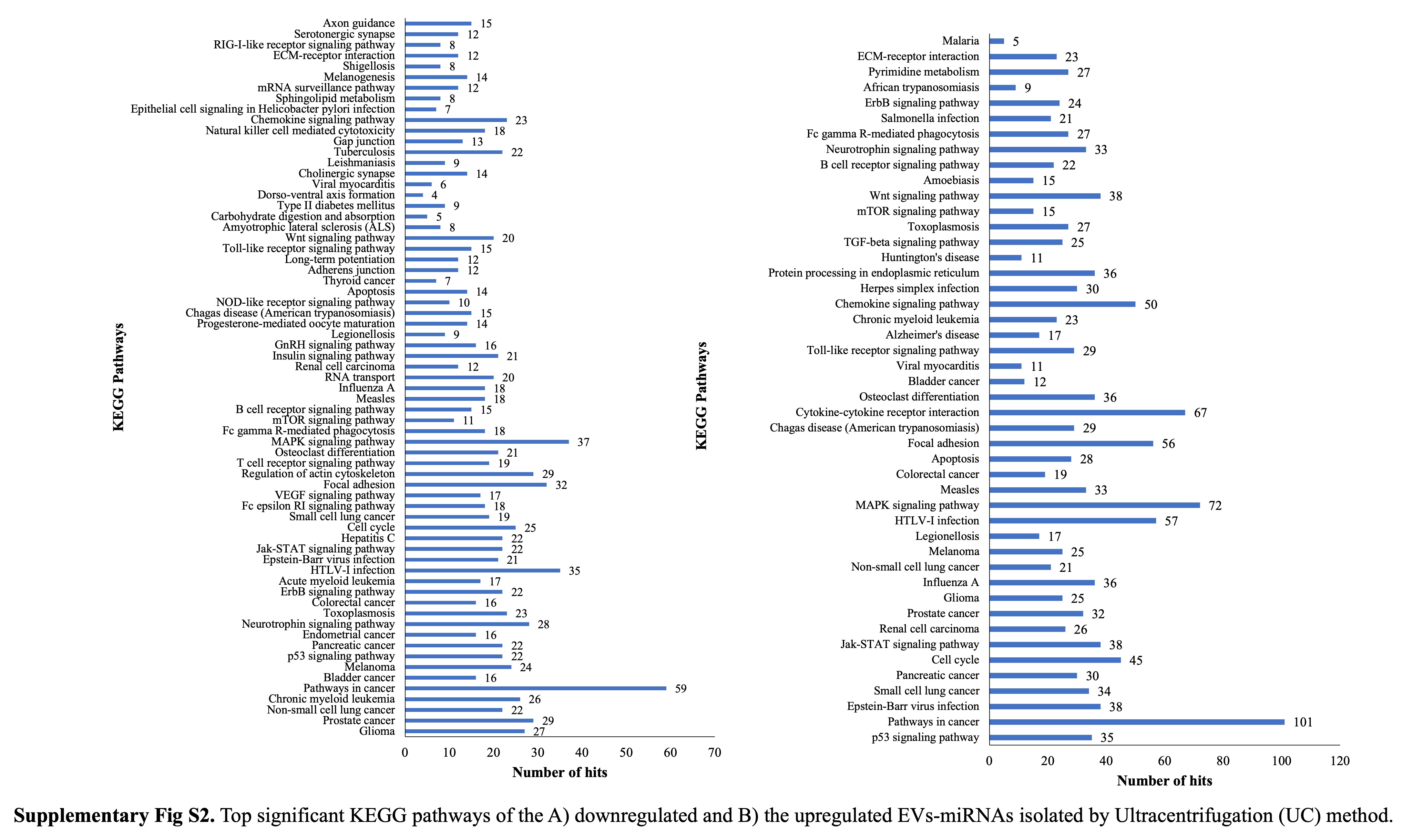

Supplement: Supplementary file 2 [file Image_2.jpg]

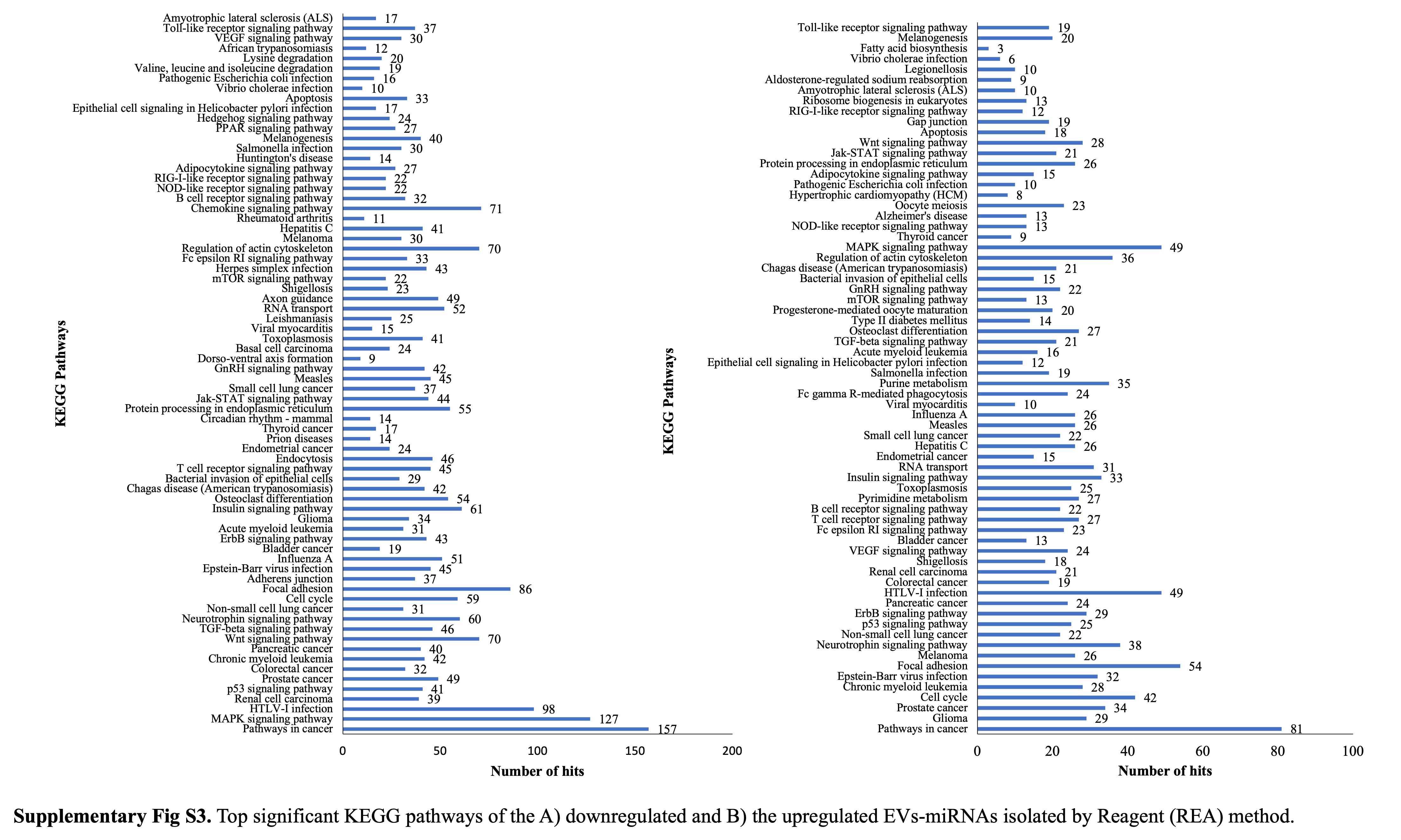

Supplement: Supplementary file 3 [file Image_3.jpg]
